# Stock Price Forecasting by a Deep Convolutional Generative Adversarial Network

**DOI:** 10.3389/frai.2022.837596

**Published:** 2022-02-04

**Authors:** Alessio Staffini

**Affiliations:** ^1^Department of Economics and Finance, Catholic University of Milan, Milan, Italy; ^2^Business Promotion Division, ALBERT Inc., Tokyo, Japan

**Keywords:** Generative Adversarial Networks, time series forecasting, stock price forecasting, deep learning, neural networks, forecasting, financial time series

## Abstract

Stock market prices are known to be very volatile and noisy, and their accurate forecasting is a challenging problem. Traditionally, both linear and non-linear methods (such as ARIMA and LSTM) have been proposed and successfully applied to stock market prediction, but there is room to develop models that further reduce the forecast error. In this paper, we introduce a Deep Convolutional Generative Adversarial Network (DCGAN) architecture to deal with the problem of forecasting the closing price of stocks. To test the empirical performance of our proposed model we use the FTSE MIB (Financial Times Stock Exchange Milano Indice di Borsa), the benchmark stock market index for the Italian national stock exchange. By conducting both single-step and multi-step forecasting, we observe that our proposed model performs better than standard widely used tools, suggesting that Deep Learning (and in particular GANs) is a promising field for financial time series forecasting.

## Introduction

The systematic practice of developing instruments for the forecasting of economic phenomena is relatively recent. Indeed, it started to become possible only in the twentieth century, as a consequence of the development of quantitative tools for analyzing the evolution of business cycles (Persons, 1916).

Modern society is characterized by the need of accurate forecasting. For example, governments want to predict the trend of many indexes, such as unemployment, inflation, industrial production, as well as the expected revenue from taxation in order to formulate effective policies. Marketing managers want to predict product demand, sales volumes, and shifts in consumer preferences in order to take appropriate decisions about current and future policies and, more generally, to formulate adequate strategic planning.

At its core, forecasting is very much linked to pattern recognition: to make a guess of what could happen in the future is based on recognizing repetitive patterns in past realizations. Of course, to make predictions based on the past is founded on the belief that the future does not reserve any significative innovation compared to what we already can observe. This is not always the case: examples are the unexpected crashes of the stock market prices around March 2020 (due to the surge of the COVID-19 pandemic) and during the financial crisis of 2007–2008. It should therefore come as no surprise that mathematical forecasting methods sometimes give poor results, even if applied correctly (Richardson, 1979).

In particular, to predict the performance of a financial stock just by observing at its previous closing prices is not a simple task. Over the years, more and more accurate programs have emerged to help in determining when to sell or buy a security, and both investment banks and listed companies now heavily rely on algorithmic trading to establish how to act on the financial markets (Gomber and Zimmermann, 2018). If we exclude the news that can influence the performance of a stock, the shift in prices is in large part affected by the conclusions that these algorithms draw from price fluctuations. In a sense, the fact that the interactions in the financial markets are increasingly guided by algorithms makes it easier to forecast the trend of the closing price of a stock (Verheggen, 2017), because algorithms act following certain patterns, and the “human factor” that could introduce heterogeneity and potential irrationality (Heiner, 1983) assumes an increasingly marginal role in decisions.

The paper is structured as follows. In Section Literature Review, we provide an overview of the literature on stock price forecasting, motivating the introduction of our model. In Section Background, we discuss the theory behind Generative Adversarial Networks. We explain our proposed model in Section The Model. In Section Empirical Analysis, we describe the empirical analysis we conducted, and we present the obtained results in Section Results. Section Conclusion concludes.

## Literature Review

The idea of rigorously analyzing stock market time series data dates back at least to 1965, when Eugene Fama analyzed if there was a correlation between stock prices over time, that is, the existence of a correlation between past and future realizations (Fama, 1965). He concluded that there was no correlation, and that each realization was random, establishing that it was not possible to simply predict stock market prices by observing their past realizations.

Over the years, Fama's conclusions have been repeatedly questioned. There are now numerous papers that try to forecast stock market prices (to name a few of them: Pai and Lin, 2005; Chang and Fan, 2008; Tsai and Wang, 2009; Sen, 2017; Khashei and Hajirahimi, 2018), using various techniques.

The most “baseline” and widely used models are undoubtedly ARIMA models. There exists a large literature of methods for forecasting stock market prices based on or originated from ARIMA (for example: Devi et al., 2013; Adebiyi et al., 2014; Banerjee, 2014; Alwadi et al., 2018).

The growth of technology allowed digital trading platforms to be born, and to perform what is called “technical analysis”, to better interpret the behavior of a stock price given its past prices.

Only recently, with the development and widespread deployment of artificial intelligence techniques, innovative models that seek to improve the performance of the existing tools have been born, especially in the context of forecasting.

The use of Artificial Neural Networks (ANNs) in developing forecasting models for financial markets gave promising results in “hybrid” models, where ANNs were combined with econometric models, such as ARIMA (Areekul et al., 2010) or GARCH (Gurusen et al., 2011). The introduction of more sophisticated neural networks designed for handling sequences of data, namely the Long Short-Term Memory (LSTM) networks, gave birth to several works that exploited their peculiarities (Roondiwala et al., 2017; Baek and Kim, 2018; Tan et al., 2019; Moghar and Hamiche, 2020). Other papers exploited Convolutional Neural Networks (CNNs) for stock price prediction (Tsantekidis et al., 2017; Hoseinzade and Haratizadeh, 2019) or Recurrent Neural Networks (RNNs) (Rather et al., 2015; Selvin et al., 2017). Researchers also used reinforcement learning techniques to build models to improve stock market trading strategies (Nevmyvaka et al., 2006).

Remaining in the field of Deep Learning, researchers have recently tried to adapt Generative Adversarial Networks (GANs) (Goodfellow et al., 2014) with the goal of analyzing and forecasting time series data. A detailed description of how GANs work is provided in Section Background but, in short, they are composed by a discriminative and a generative network that interact with each other: the former trying to distinguish whether a certain instance is real or fake, and the latter trying to confuse the discriminative network by generating increasingly realistic data. In 2018, Zhou et al. (2018) developed a GAN that used an LSTM as a generator and a CNN as a discriminator to forecast the high-frequency stock market. In the same year, Luo et al. (2018) proposed a similar model for predicting crude oil prices. The next year, Koochali et al. (2019) introduced a conditional GAN to compute probabilistic forecasts on web traffic data; again, in 2019, Zhang et al. proposed a GAN with a Multi-Layer Perceptron (MLP) acting as a discriminator for forecasting the closing price of some stocks of S&P 500 Index and PAICC, among others. All these works compared the obtained performances with those of other machine learning and time series models, such as LSTM and ARIMA, obtaining promising results. In 2020, Zhou et al. (2020a) compared their GAN with other models (such as ARIMA, Temporal Convolutional Network, and LSTM) by testing them on public benchmark datasets, concluding that the generative network achieved comparable forecasting accuracy with the other methods. In the same year, Zhou et al. (2020b) published another work on a web traffic dataset, further confirming that GAN results were neither better nor worse than the other considered models. On the other hand, Lin et al. (2021) obtained superior results using GANs but evaluated the considered models over the time series of a single stock (Apple Inc.).

In recent years, GANs have shown promising results in solving many complex problems (e.g., realistic image and video generation, image-to-image and text-to-image translation) but to show that they can provide better results also for financial time series forecasting (compared to more traditional approaches) remains a challenge yet to be solved.

The contribution of the present paper is 2-fold. First, we propose a novel and stable deep convolutional GAN architecture, both in the generative and discriminative network, for stock price forecasting. Second, we compare and evaluate the performance of the proposed model on 10 heterogeneous time series from the Italian stock market. To the best of our knowledge, this is the first GAN architecture applied to stock price forecasting that uses convolutional layers both in the generator and in the discriminator. We also propose a modification to the loss function of the generator, adding further terms that improves the forecasting process. Furthermore, as highlighted in Section Results, our proposed model is much more stable (in the sense that the standard deviation of the results is lower) compared to previously proposed GAN architectures for time series forecasting.

## Background

Generative Adversarial Networks are a class of generative models that has shown remarkable results in many tasks, in particular image generation and image-to-image translation. In its original formulation, a GAN is composed by two neural networks (the generator *G* and the discriminator *D*) that compete against each other in a zero-sum non-cooperative game. The generator produces “fake” samples by mapping a vector of random noise *z*∈*Z*, *z* ~ *p*(*z*), where *Z* denotes the latent space, and *p*(*z*) is the random noise distribution (typically a uniform or a Gaussian distribution). The goal of the generator is to fool the discriminator into believing that the generated sample is real (i.e., it wants to capture the characteristics of the real data distribution), while the discriminator acts as a classifier that must distinguish between fake and real data samples, outputting a scalar *D*(*a*)∈[0, 1], which can be understood as the probability that the discriminator assigns to the sample *a* to belong to the real distribution. More formally, *G* and *D* are trained by playing the following minimax game with value function *V*(*G, D*):


(1) $$\begin{array}{l} \underset{G}{\text{min}}\underset{D}{\text{ max }}V\left(G,D\right)=\;E_{x\sim p\left(r\right)}\left[\log\left(D\left(x\right)\right)\right]\\ +E_{z\sim p\left(z\right)}\left[\log\left(1-D\left(G(z\right)\right)\right], \end{array}$$

where *p*(*r*) denotes the real data distribution of the samples *x*. If we denote with $\tilde{x}=G\left(z\right)$ the output of the generator, we can rewrite Equation (1) as:


(2) $$\begin{array}{l} \underset{G}{\text{min}}\underset{D}{\text{ max }}V\left(G,D\right)=\;E_{x\sim p\left(r\right)}\left[\log\left(D\left(x\right)\right)\right]\\ +E_{\tilde{x}\sim p\left(g\right)}\left[\log\left(1-D\left(\tilde{x}\right)\right)\right], \end{array}$$

where *p*(*g*) is the generator's distribution over data *x*. We can appreciate the adversarial nature of the game by noticing that, in the second term of Eq. 2, the discriminator cares about correctly classifying fake samples, while on the other hand the generator wants it to classify them as true. During training, each network forces the other to improve. If we denote with θ^*D*^ and θ^*G*^ the parameters of the discriminator and the generator respectively, the update at iteration *n*+1 is described by the following two rules:


$$\begin{array}{r} \theta_{n+1}^{D}\leftarrow \;\theta_{n}^{D}+\eta_{D}\left(\nabla_{\theta^{D}}\frac{1}{m}\;\overset{m}{\underset{i=1}{\sum }}\left[\log\left(D\left(x^{i}\right)\right)+\log\left(1-D\left({\tilde{x}}^{i}\right)\right)\right]\right),\\ \theta_{n+1}^{G}\leftarrow \;\theta_{n}^{G}-\eta_{G}\left(\nabla_{\theta^{G}}\frac{1}{m}\;\overset{m}{\underset{i=1}{\sum }}\left[\log\left(1-D\left({\tilde{x}}^{i}\right)\right)\right]\right), \end{array}$$

that are calculated by sampling *m* real and fake samples, where η_*D*_>0 and η_*G*_>0 are the learning rates of the discriminator and the generator, respectively.

**Figure 1:**
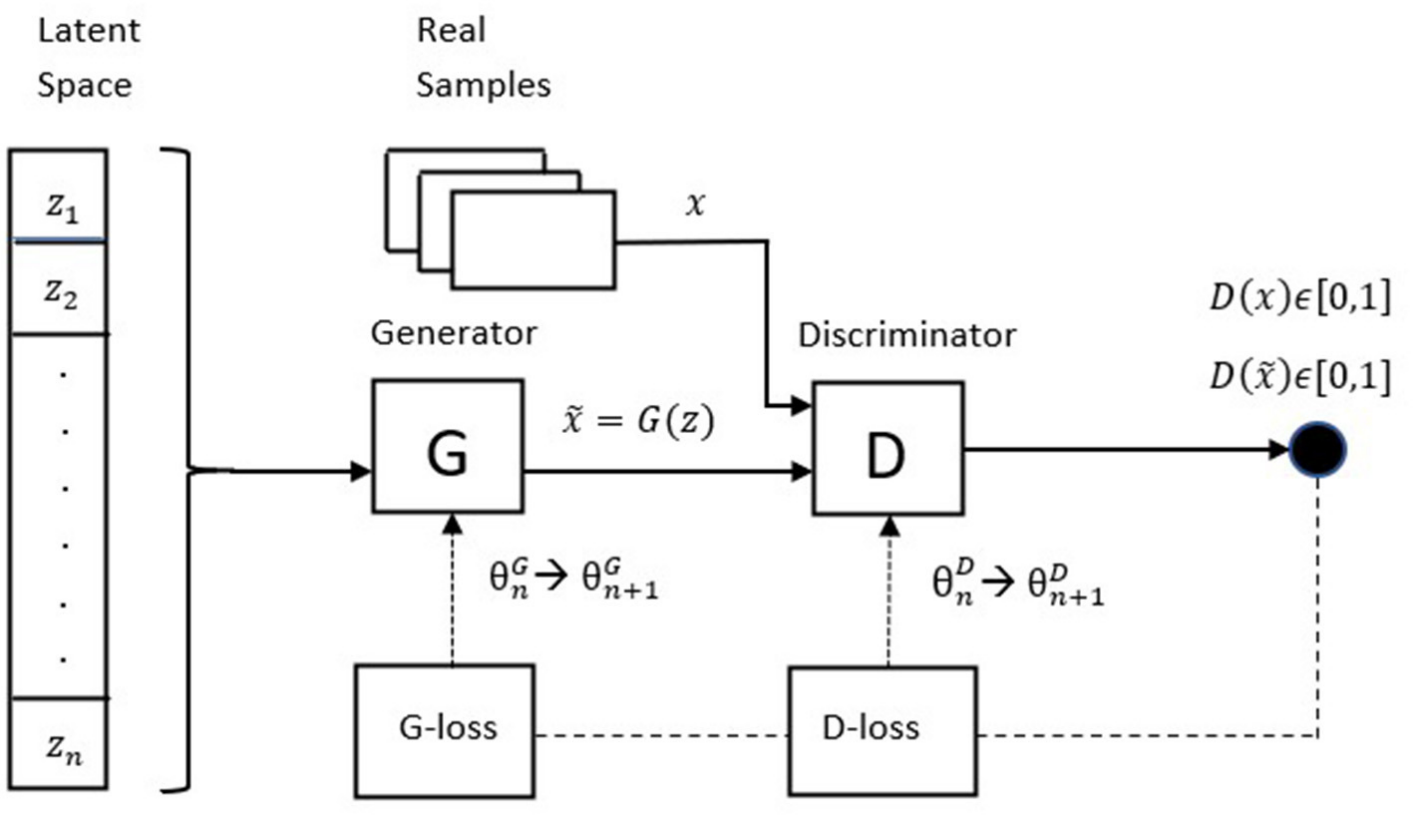
Stylized architecture of the original GAN.

Figure 1 shows the architecture of the described GAN.

The training process is supposed to continue until Nash equilibrium, that is, when $D\left(x\right)=D\left(\tilde{x}\right)=0.5$ and *p*(*g*) = *p*(*r*). In practice, reach equilibrium in non-convex games is difficult^1^. Without reaching equilibrium GANs can nevertheless produce good results, despite existing however a number of other unresolved problems with the original GAN implementation: in particular, vanishing gradients (when the discriminator does not provide a useful feedback) and mode collapse (when the generator finds how to trick the discriminator by producing samples with low variety).

In their paper, Goodfellow et al. (2014) showed that if the discriminator is trained to optimality before each update in the generator, then by minimizing *V*(*G, D*) the generator is minimizing the Jensen-Shannon (JS) divergence between *p*(*r*) and *p*(*g*). However, it can be shown that the JS divergence induces a strong topology, and it might be discontinuous, often leading in applications to the vanishing gradient problem since it is difficult to maintain the discriminator at the same level of the generator for the whole training process.

To overcome this problem, Arjovsky et al. (2017) introduced a variant of the original GAN based on the Wasserstein distance (WGAN), which is shown to induce a weaker topology and it is a more sensible cost function when we care about convergence in distribution. The Wasserstein-1 distance (also known as Earth-Mover distance) between *p*(*r*) and *p*(*g*) is defined as:


(3) $$\begin{array}{l} W\left(p\left(r\right),p\left(g\right)\right)=\underset{\gamma \epsilon \Pi \left(p\left(r\right),p\left(g\right)\right)}{\inf}E_{\left(x,\tilde{x}\right)\sim \gamma }\left[\left||x-\tilde{x}|\right|\right], \end{array}$$

where Π(*p*(*r*), *p*(*g*)) denotes the set of all possible joint distributions of *p*(*r*) and *p*(*g*), and $\gamma \left(x,\tilde{x}\right)$ informally denotes how much “mass” has to be transported from point *x* to point $\tilde{x}$ in order to transform the fake distribution *p*(*g*) into the real distribution *p*(*r*). *W*(*p*(*r*), *p*(*g*)) is continuous everywhere and differentiable almost everywhere, leading to higher stability in training and a non-saturating discriminator. However, it is computationally intractable to consider all the possible joint distributions γ* ϵ Π*(*p*(*r*), *p*(*g*)) to compute the infimum. By applying the Kantorovich-Rubinstein duality, we can rewrite Equation (3) as:


(4) $$\begin{array}{l} W\left(p\left(r\right),p\left(g\right)\right)=\underset{||F|{|}_{L}\leq 1}{\sup}E_{x\sim \;p\left(r\right)}\left[F\left(x\right)\right]-E_{\tilde{x}\sim \;p\left(g\right)}\left[F\left(\tilde{x}\right)\right], \end{array}$$

where the supremum is taken over F, the set of 1-Lipschitz continuous functions^2^. Since now the loss function measures the Wasserstein distance between *p*(*r*) and *p*(*g*), the discriminator in Equation (4) takes up the role of learning an appropriate function to compute such distance, rather than directly discriminating between fake and real samples. The minimax game is now defined by:


(5) $$\begin{array}{l} \underset{G}{\text{min}}\underset{D\in F}{\text{ max }}V\left(G,D\right)=E_{x\sim p\left(r\right)}\left[D\left(x\right)\right]-E_{\tilde{x}\;\sim p\left(g\right)}\left[D\left(\tilde{x}\right)\right], \end{array}$$

where the notation is consistent with above. However, in order for the discriminator's gradient to be informative, we have to enforce 1-Lipschitz continuity on the discriminator. In their paper, Arjovsky et al. (2017) did so by clipping the weights of the discriminator, constraining them within a compact space, but the authors themselves mentioned that such approach is not optimal and might lead to training instability.

A further improvement on the WGAN architecture moved in the direction of finding a more suitable way to enforce Lipschitz constraints. Gulrajani et al. (2017) proposed using gradient penalty (WGAN-GP) to deal with the problem, exploiting the fact that a function *f* is 1-Lipschitz if and only if ||∇*f*|| ≤ 1 everywhere. They added a further penalty term to the value function defined in Equation (5), which acts as a regularization by penalizing ||∇*D*||≠ 1:


(6) $$\begin{array}{l} \underset{G}{\text{min}}\underset{D\in F}{\text{ max }}\;V\left(G,D\right)=E_{x\sim p\left(r\right)}\left[D\left(x\right)\right]-E_{\tilde{x}\sim p\left(g\right)}\left[D\left(\tilde{x}\right)\right]\\ -\lambda E_{\hat{x}\;\sim p\left(i\right)}\left[{\left(\left\|\nabla_{\hat{x}}D(\hat{x})\right\|-1\right)}^{2}\right], \end{array}$$

where λ is a hyperparameter (Gulrajani et al., 2017 observed λ = 10 to work empirically well on a wide number of tasks) that controls the tradeoff between the new regularization term and the other terms. Since enforcing the unit gradient norm everywhere is intractable, the authors considered sampling interpolated points $\hat{x}$ between the real samples *x* and the fake samples $\tilde{x}$:


$$\begin{array}{l} \hat{x}=\;\alpha x+\left(1-\alpha \right)\tilde{x}, \end{array}$$

with α~*U*[0, 1], $\hat{x}\sim p\left(i\right)$. Empirically, the added regularization term works well, and WGAN-GP is less prone to mode collapse and vanishing gradient problems than other architectures. More explicitly, from *V*(*G, D*) in Equation (6) we can separate the loss functions for the discriminator and the generator in WGAN-GP:


(7a) $$\begin{array}{l} L_{D}=E_{\tilde{x}\sim p\left(g\right)}\left[D\left(\tilde{x}\right)\right]-E_{x\sim p\left(r\right)}\left[D\left(x\right)\right]\\ \;+\lambda E_{\hat{x}\sim p\left(i\right)}\left[{\left(||\nabla_{\hat{x}}D\left(\hat{x}\right)||-1\right)}^{2}\right], \end{array}$$

(7b) $$\begin{array}{l} L_{G}=-E_{\tilde{x}\sim p\left(g\right)}\left[D\left(\tilde{x}\right)\right], \end{array}$$

where *L*_*D*_ and *L*_*G*_ denote the loss functions of the discriminator and the generator, respectively.

## The Model

We propose using a Deep Convolutional Generative Adversarial Network (DCGAN) for the task of accurately forecasting the stock price evolution. The idea behind why we use an adversarial network is because we want to mimic the learning process of a financial trader: using a set of available predictors, the unexperienced trader (the generator in the first epochs) makes certain predictions about the stock price, which are then progressively corrected by looking at the real realizations (the work of the discriminator), until he/she becomes experienced (the generator in the last epochs).

Since GANs are mainly used for generative modeling, the generator traditionally receives in input a random vector *z* from the latent space, and thanks to the learning process it tunes its own parameters to map that vector into a realistic object (very often, an image). However, when dealing with time series forecasting, it makes more sense to provide as input to the generator the matrix of predictors $X=\left[\begin{array}{cccc} x_{1,1} & x_{2,1} & \ldots & x_{i,1}\\ x_{1,2} & x_{2,2} & \ldots & x_{i,2}\\ \vdots & \vdots & \ddots & \vdots \\ x_{1,t} & x_{2,t} & \ldots & x_{i,t} \end{array}\right]$, where we include *i* variables observed over *t* time steps, and use *X* to forecast the value in *t*+1 of the variable of interest *y* (which might or might not be a variable included in *X*). We can think of the problem as wanting the generator to learn the data distribution of *y*_*t*+ 1_.

On the other hand, as for the data input for the discriminator, we constructed the fake samples by adding the generator's output to the real data sequence, ỹ = {*y*_1_, *y*_2_, …, *y*_*t*_, ỹ_*t*+1_}, and the real samples by adding the true realization, *y* = {*y*_1_, *y*_2_, …, *y*_*t*_, *y*_*t*+1_}. Following Zhang et al. (2019), the idea of providing sequences to the discriminator instead of just ỹ_*t*+1_ and *y*_*t*+1_ is because we want it to also extract and learn useful information on the correlation over time steps.

There are also other two important aspects that need to be mentioned. First, following Chintala (2016), we normalized the data to lie within [−1, 1]. This also has the benefits of bringing all the predictors to the same range. Second, following the WGAN paper, we trained the discriminator five times more than the generator, during each training iteration. The idea behind this is that in WGAN the discriminator will not saturate, so the more we train it the closer to optimality we get, obtaining therefore more reliable gradient information for the training process.

Due to their useful properties, we used both the Wasserstein distance and gradient penalty as suggested in the WGAN-GP paper but, since we are dealing with time series, we modified the loss function of the generator (Equation 7b) by including further information to “guide” the generator into producing good samples:


(8a) $$\begin{array}{l} L_{D}=\frac{1}{m}\overset{m}{\underset{i=1}{\sum }}\left[D\left({ỹ}^{i}\right)\right]-\frac{1}{m}\overset{m}{\underset{i=1}{\sum }}\left[D\left(y^{i}\right)\right]\\ \;+\lambda \frac{1}{m}\;\overset{m}{\underset{i=1}{\sum }}\left[{\left(||\nabla_{ŷ}D\left({ŷ}^{i}\right)||-1\right)}^{2}\right]\;, \end{array}$$

(8b) $$\begin{array}{l} L_{G}=\;-\frac{1}{m}\overset{m}{\underset{i=1}{\sum }}\left[D\left({ỹ}^{i}\right)\right]+\psi_{1}\frac{1}{m}\overset{m}{\underset{i=1}{\sum }}{\left(y^{i}-{ỹ}^{i}\right)}^{2}\\ \;+\psi_{2}\frac{1}{m}\overset{m}{\underset{i=1}{\sum }}|\text{sgn}\left(y^{i}\right)-\text{sgn}\left({ỹ}^{i}\right)|, \end{array}$$

where the two losses in Equations (8a) and (8b) are computed over a batch of *m* samples. The second term in the generator loss computes the MSE (penalizing large errors between the real and fake samples), while the third term further guides the generator into producing fake samples that have the same sign (i.e., that are close to) the real samples (recall that we normalized the data to range between −1 and 1). ψ_1_ and ψ_2_ are two hyperparameters that weight the importance of these added loss components, and we found ψ_1_ = ψ_2_ = 0.5 to work well across the considered time series, but some tuning might be necessary for different applications. Notice that, at least theoretically, these added components are not strictly necessary: if *D*(.) is trained to be close to optimality in each epoch, we could in principle use only its gradient to update in a reliable way the generator. However, empirically we found that these added loss components significatively speeded up the training process, requiring less epochs to convergence (after 300 epochs, the measured loss was half the one observed in the standard formulation). We trained our architecture for 1,200 epochs.

For the generator network, we selected a CNN-BiLSTM architecture. First, we stacked a one-dimensional convolutional layer (32 filters, with a filter size of 2) to process the input values. Long Short-Term Memory networks (LSTMs) (Hochreiter and Schmidhuber, 1997) have been proven to be very effective in dealing with sequence data, so the results obtained from the convolutional layer are passed to a Bidirectional-LSTM (BiLSTM) layer with 64 units, the bidirectionality being useful in order to provide more context for the forecasting process. Finally, we stacked 2 fully connected layers of 64 and 32 neurons, respectively. The output layer is composed by a single neuron when we are forecasting only the next time step, and 5 neurons when we are forecasting the next 5 days.

For the discriminator network, we selected a CNN architecture, which is appropriate when we have to compare and discern two types of sequences (real and fake). To avoid possible imbalances while training, it is useful to have a symmetric architecture between the generator and the discriminator. Therefore, we stacked 2 one-dimensional convolutional layers (32 and 64 filters each, with a filter size of 2), followed by 2 fully connected layers of 64 and 32 neurons, respectively. Since the discriminator has to output a single scalar that represents how real the input sequence is, the output layer was made by a single neuron without any activation function.

Regarding the activation functions, we applied Leaky Rectified Linear Unit (Leaky ReLU) to all the hidden layers in the generator (except the BiLSTM layer that uses just standard ReLU) and in the discriminator. Its equation is given by:


$$\begin{array}{l} LeakyReLU\left(x\right)=\{\begin{array}{ll} ax & x<0\\ x & x\geq 0 \end{array} \end{array}$$

where we set α = 0.1. To allow for a positive slope in the negative region is helpful when we might suffer from sparse gradients, as in GANs training.

To avoid overfitting and obtain greater regularization, we applied Dropout (Srivastava et al., 2014) in each hidden layer of the generator and the discriminator, with the exception of convolutional layers, where it is less effective. Since the number of neurons is not too high, we found a moderate dropout rate *p* = 0.2 to work well in practice (in the BiLSTM layer we applied *p* = 0.3).

We initialized the weights of the two networks by using a zero-centered Gaussian distribution with standard deviation 0.02, as in Radford et al. (2015). As for the optimization algorithm, we selected Adam (Kingma and Ba, 2014), but we tuned its hyperparameters to obtain more stability in training: in particular, we found that in addition to a higher number of discriminator iterations per generator iteration, it was also helpful to set a higher learning rate for the discriminator. Therefore, we set the learning rate of the discriminator η_*D*_ to 0.0004, and the learning rate of the generator η_*G*_ to 0.0001. Following Radford et al. (2015), we lowered the momentum term β_1_ to 0.5, but we also lowered β_2_ to 0.9.

**Figure 2:**
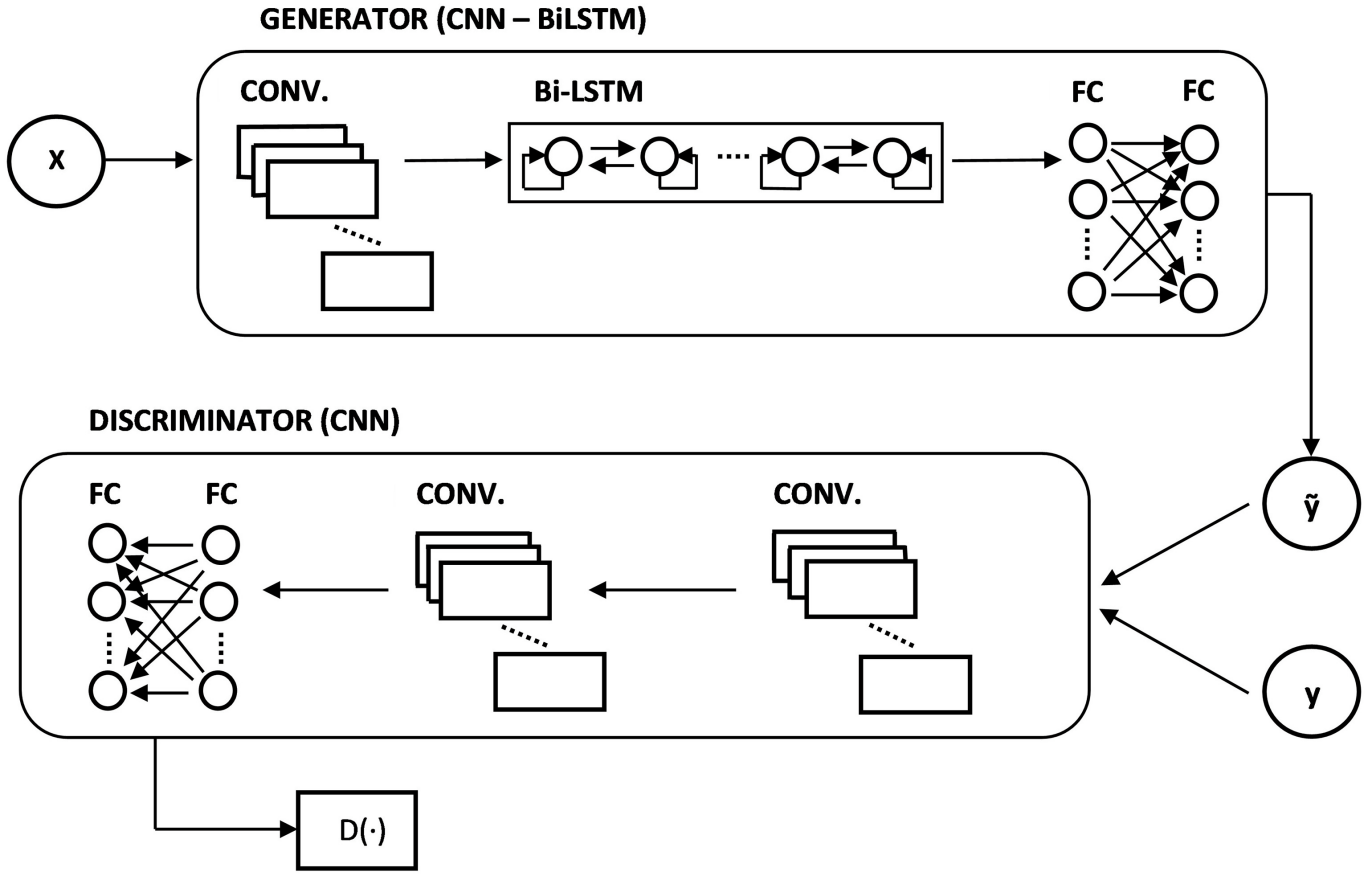
Stylized architecture of our DCGAN.

Figure 2 shows the architecture of our GAN.

## Empirical Analysis

We conducted our empirical analysis starting from the time series data of closing, opening, maximum and minimum prices, as well as the daily trading volume of the stocks from the FTSE MIB (Financial Times Stock Exchange Milano Indice di Borsa) Index. The data are available online (https://it.finance.yahoo.com/). To train deep learning models successfully a “high enough” number of observations is required, and so we chose to include the stocks which values started from January 2005. We selected 26 stocks with these characteristics (starting from the original 40 in FTSE MIB). From this initial selection, we selected 10 stocks out of 26 (see Table 1) on which perform our analysis. The selection criteria for these 10 stocks were the heterogeneity (i.e., to include companies from different sectors) and the time series behaviors (i.e., we included stocks that did not display a clear tendency to increase or decrease, over the considered period of time).

**Table 1:** The 10 selected stocks.

**Company**	**Ticker**	**Sector**
Atlantia	ATL	Industrial Transportation
Azimut	AZL	Financial Services
Buzzi Unicem	BZU	Construction & Materials
Enel	ENEL	Electricity
Eni	ENI	Oil & Gas Producers
Exor	EXO	Financial Services
Generali	G	Non-life Insurance
Interpump Group	IP	Industrial Goods & Services
Mediobanca	MB	Banks
Recordati	REC	Pharmaceuticals & Biotechnology

The time series for each stock is composed of 4,149 observations, from January 2005, 3rd to April 2021, 30th. Regarding data preprocessing, the missing values were very few for each stock, ranging from a maximum of 18 to a minimum of 0, with an average of 11.27 (0.27% of total length). We replaced those missing values with the previous observation in the time series.

Starting from these data, we constructed other variables that are often used for the technical analysis of stocks, as well as to forecast their future prices. Technical analysis has often been criticized for its reliance on past data and for ignoring the market fundamentals; however, technical indicators are still widely used for stock price forecasting and have been proven to be effective in many works (e.g., Oriani and Coelho, 2016; Aras, 2020; Agrawal et al., 2021). To keep a simple framework (technical indicators can be readily used by non-experts) and to compare our results with those of other works, we decided to use these indicators only, well-knowing that traders do not rely on technical analysis only when making investment choices.

The total list of the variables we used is reported in Table 2.

**Table 2:** List of the variables we considered for the empirical analysis.

**Variable**	**Description**
Close	Closing price of the stock at the end of day
Closed	Difference in the stock price compared to the previous close
Closep	Percentage change in the stock price compared to the previous close
Ope	Opening price of the stock
Oped	Difference in the stock price compared to the previous opening
opep	Percentage change in the stock price compared to the previous opening
high	Highest price reached by the stock on the trading day
highd	Difference in the highest price reached by the stock compared to the previous day
highp	Percentage change in the highest price achieved by the stock compared to the previous day
low	Lowest price reached by the stock on the trading day
lowd	Difference in the lowest price reached by the stock compared to the previous day
lowp	Percentage change in the lowest price reached by the stock compared to the previous day
SMA5	Simple Moving Average of the closing price calculated at 5 days
SMA10	Simple Moving Average of the closing price calculated at 10 days
EMA12	Exponential Moving Average of the closing price calculated at 12 days
EMA26	Exponential Moving Average of the closing price calculated at 26 days
MACD	Moving Average Convergence/Divergence. It is a technical indicator to reveal changes in the strength, direction, momentum, and duration of a trend. It is calculated as the difference between the EMA26 and EMA12
MACDsign	The exceeding of MACD values on MACDsign and vice versa are useful signals to identify possible trend reversals in prices. It is calculated as a 9-period exponential moving average of the MACD line
volume	Number of shares traded on the day
volumed	Difference in the number of traded shares compared to the previous day
volume	Percentage change in the number of traded shares compared to the previous day
RoC13	Rate of Change. The ROC calculates the ratio of today's closing price to the closing price of (in our case) 13 previous days
K15	Stochastic Oscillator. The Stochastic Oscillator compares the closing price of the stock with the range of prices in the considered time period (15 days, in our case)
D5	Simple Moving Average of the values of the variable K15 calculated at 5 days
SMA20C	Simple Moving Average of the closing price calculated at 20 days
BOLlow	Bottom line of the Bollinger Bands
BOLup	Upper line of the Bollinger Bands
BOL	Percentage BOL, a volatility index of the stock. It is constructed as the ratio between the difference of close and BOLlow with the difference between BOLup and BOLlow
MOM12	Momentum, an indicator that measures the change in closing prices. Unlike the ROC, the momentum is calculated as the difference between today's closing price and the closing price of the previous period (12 days, in our case)

**Figure 3:**
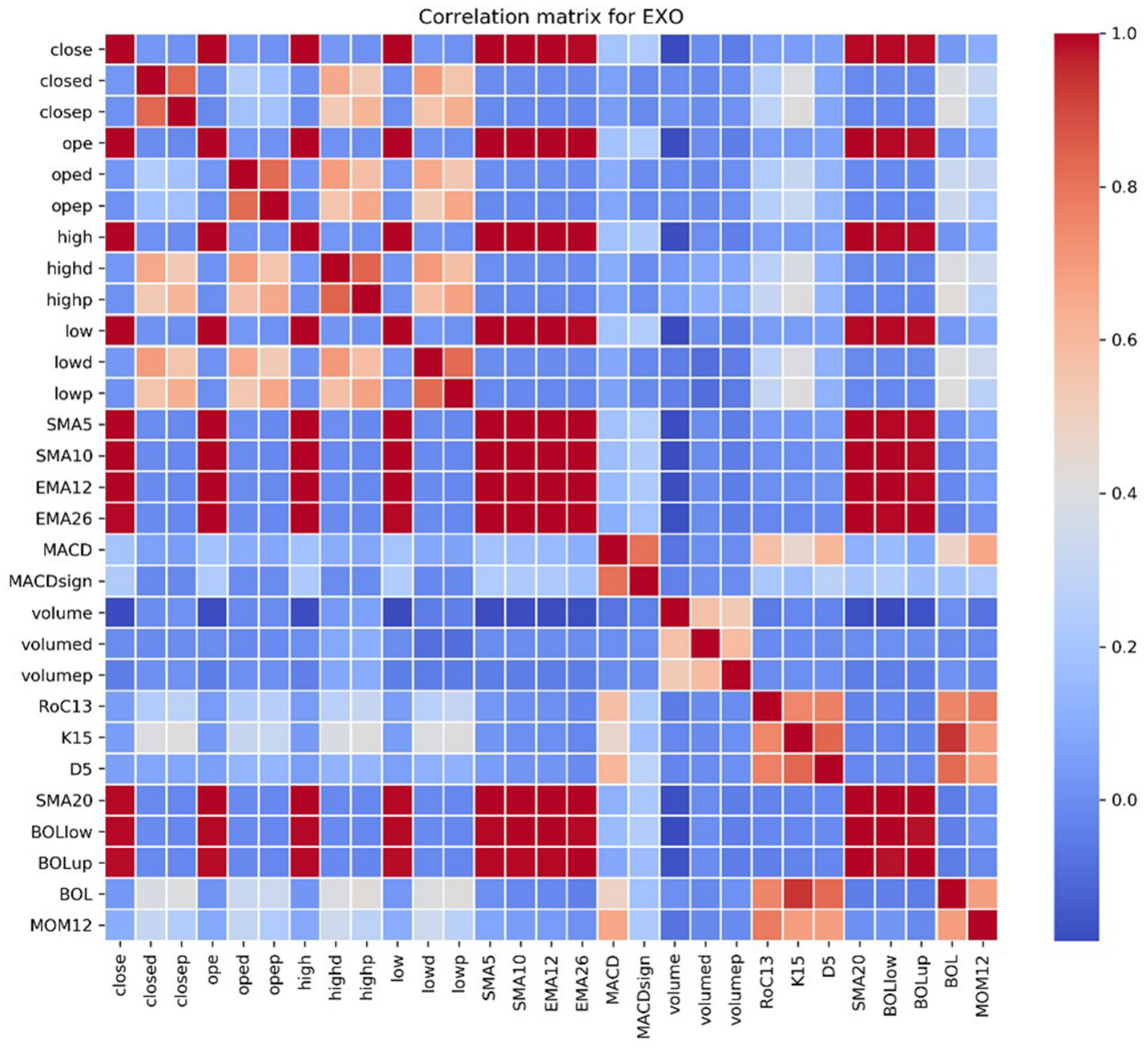
Correlation matrix for EXO of the considered technical indicators.

For each stock, we checked the correlation matrix of the selected variables, and we observed how many variables have a correlation close to 0 with the closing price. Figure 3 shows the correlation matrix for EXO.

Furthermore, even if we selected stocks that did not exhibit clear trends, by performing the Augmented Dickey-Fuller (ADF) test (Dickey and Fuller, 1979) we could not rule out the existence of unit roots for the closing price, the opening price, the highest and the lowest price variables at significance level 0.01. Hence, when we ran the ARIMAX models (see Section Results), we took the first differences for these variables (the ADF test performed on the first differences rejected the presence of unit roots at significance level 0.01).

Our goal is to forecast the values of the closing price of the stocks, and among the technical indicators we constructed we therefore selected a subset of them that are meaningful and strongly correlated with the dependent variable, across the 10 selected stocks: ope, high, low, SMA5, SMA10, SMA20, EMA12, EMA26, MACD, MACDsign, volume, BOLlow, and BOLup.

**Figure 4:**
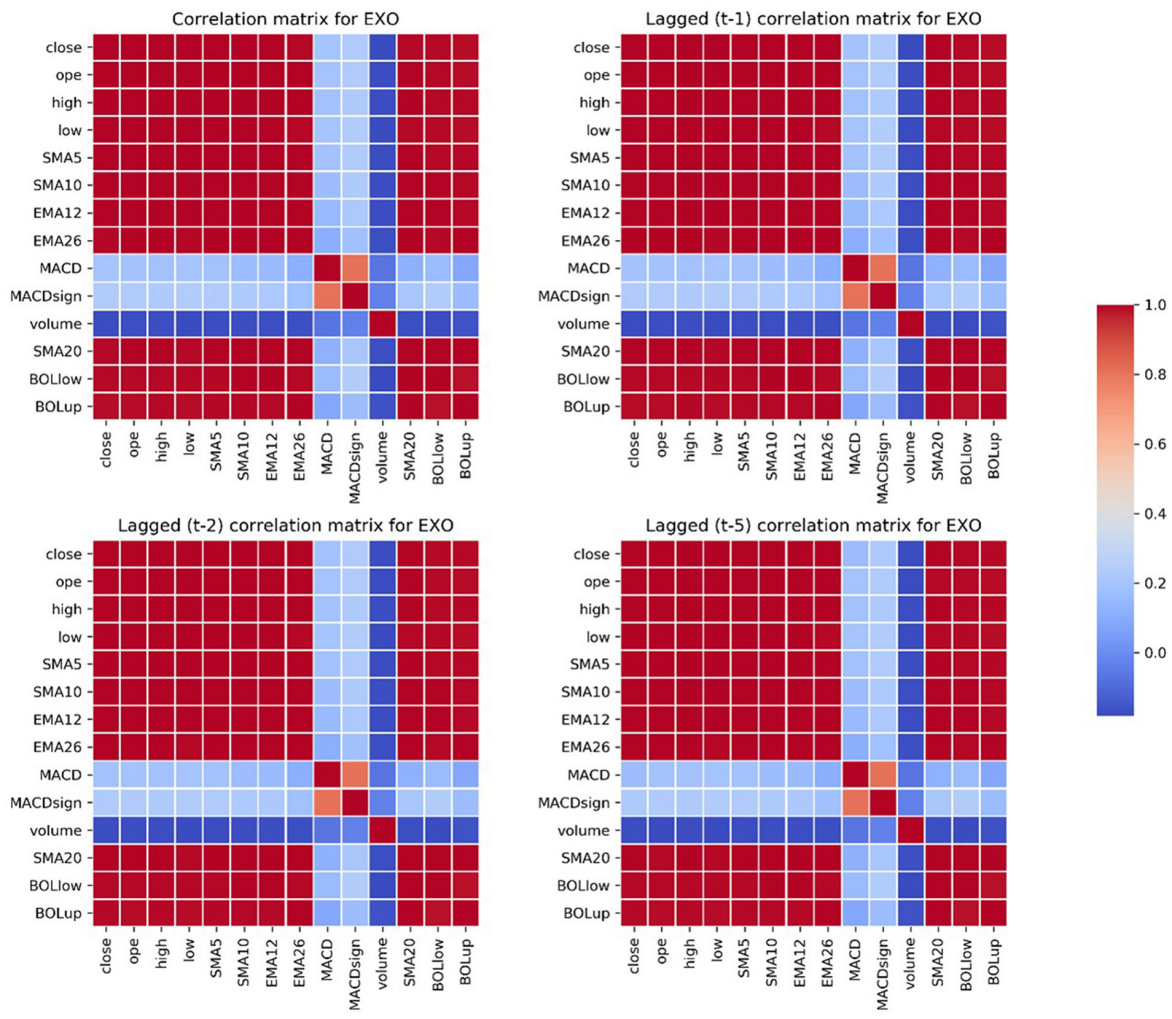
Contemporaneous and lagged correlation matrices for EXO of the selected variables.

Figure 4 shows the correlation matrices of the subset of regressors we considered for the analysis (at time *t*, *t*−1, *t*−2, and *t*−5) with the closing price (at time *t*), for the case of EXO. We can observe that the correlation matrices do not vary when we consider lagged values, indicating that the variables selection is robust.

## Results

To evaluate the performance of our GAN architecture we compared its results with those of four widely used models for stock price forecasting: ARIMAX-SVR, Random Forest Regressor, LSTM, and a benchmark GAN.

ARIMAX (Autoregressive Integrated Moving Average with Extra Input) is an extension of ARIMA, including not only the lagged values of the dependent variables but other variables as well as predictors. Considering that we want to model *y*_*t*_ by including not only its *p* lagged observations and the *q* lagged observations of the residual error terms, but also the *p* lagged observations of *n* further predictors, the equation of an *ARIMAX*(*p, d, q, n*) is given by:


$$\begin{array}{l} {\Delta_{d}y}_{t}=\beta_{0}+\;\overset{p}{\underset{i=1}{\sum }}\beta_{i}{\Delta_{d}y}_{t-i}+\overset{n}{\underset{s=1}{\sum }}\overset{p}{\underset{k=1}{\sum }}\varphi_{k}^{\left(s\right)}x_{t-k}^{\left(s\right)}+\;\varepsilon_{t}+\overset{q}{\underset{j=1}{\sum }}\theta_{j}\varepsilon_{t-j}, \end{array}$$

where $B={\left(\beta_{1},\beta_{2},\;\ldots ,\;\beta_{p}\right)}^{T}$ is the vector of coefficients of the lagged values of the dependent variable, *X* = (*x*^(1)^, *x*^(2)^, …, *x*^(*n*)^)^*T*^ is the vector of extra predictors,$\Phi^{\left(s\right)}={\left(\varphi_{1}^{\left(s\right)},\varphi_{2}^{\left(s\right)},\;\ldots ,\;\varphi_{p}^{\left(s\right)}\right)}^{T}$ is the vector of coefficients of the extra predictor *s*, with *n, p*>0. Δ_*d*_ denotes the *d*-th difference of *y*_*t*_, and β_0_ is the intercept of the model (i.e., the mean of the series);$\varepsilon_{t}\sim WN\left(0,\sigma^{2}\right)$ denotes the error term and $\Theta ={\left(\theta_{1},\theta_{2},\;\ldots ,\;\theta_{q}\right)}^{T}$ is the vector of lagged error terms coefficients, *q*>0. In the context of our analysis, the *n* further predictors are those strongly correlated with the closing price of the stocks (see Figure 4), so that *n* = 13. As explained in Section Empirical Analysis, we could not rule out that some considered predictors where *I*(1) processes; hence, we set *d* = 1, and a further ADF test on the differentiated data of these variables confirmed the absence of unit roots. For finding the most suitable order of *p* and *q*, we analyzed the autocorrelation function (ACF) and the partial autocorrelation function (PACF). We further compared the model specification suggested by ACF and PACF with the one suggested by the Akaike Information Criterion (AIC) (Akaike, 1973). The specifications suggested by ACF/PACF and AIC often matched; when they did not, we preferred the ones that empirically performed better.

One of the main features of financial time series is volatility clustering (conditional heteroskedasticity). To this end, following Pai and Lin (2005), we improved the forecasts obtained with the ARIMAX model by augmenting it with a Support Vector Regression (SVR) backend, in order to capture the non-linear patterns in the data. More specifically, denoting with ε_*t*_ the residual of the ARIMAX model at time *t*, we modeled it as follows:


$$\begin{array}{l} \varepsilon_{t}=\;f\left(\varepsilon_{t-1},\varepsilon_{t-2},\;\ldots ,\;\varepsilon_{t-n}\right)+\;u_{t}, \end{array}$$

with *f* being a non-linear function defined by the SVR model, which takes as input past realizations of the ARIMAX residuals, and *u*_*t*_ being a random error. We used the radial basis function as kernel for the SVR algorithm. The number of inputs, as well as the kernel coefficient, the regularization parameter and the epsilon-tube parameter were optimized for each stock on the training data using a grid search approach.

The Random Forest (RF) Regressor is a non-linear machine learning algorithm which uses ensemble learning, constructing a group of decision trees and aggregating their predictions to tackle regression problems. Although typically not used for time series forecasting, it has proved to be successful in this area as well, and it is recently gaining popularity (Kane et al., 2014; Dudek, 2015; Masini et al., 2021). The number of inputs, as well as the number of decision trees in the forest and the number of features to consider when looking for the best split were optimized for each stock on the training data using a grid search approach. Since in RFs there is randomness coming both from bootstrapping and the sampling of the features to consider when looking for the best split at each node, in our experiments we considered the results over 10 different seeds.

LSTMs are a class of deep learning models, thought especially to deal with sequence data and to deal with the vanishing problem of RNNs. In particular, each LSTM cell contains three gates: a forget gate (which establishes what information should be discarded), an input gate (to establish which values from the input should be used to update the memory state), and an output gate (which decides what to output based on the input and the memory cell). The gates can be understood as weighted functions, and their parameters are updated using Backpropagation Through Time (BPTT) (Mozer, 1989). The LSTM cell equations at a given time step *t* are as follows:


$$\begin{array}{lll} F_{t} & = & \sigma \left(W_{F}x_{t}+U_{F}h_{t-1}+b_{F}\right),\\ I_{t} & = & \sigma \left(W_{I}x_{t}+U_{I}h_{t-1}+b_{I}\right),\\ {\tilde{C}}_{t} & = & \tanh\left(W_{C}x_{t}+U_{C}h_{t-1}+b_{C}\right),\\ O_{t} & = & \sigma \left(W_{O}x_{t}+U_{O}h_{t-1}+b_{O}\right),\\ C_{t} & = & F_{t}\circ C_{t-1}+I_{t}\circ {\tilde{C}}_{t},\\ h_{t} & = & O_{t}\circ \tanh\left(C_{t}\right), \end{array}$$

where *F*_*t*_, *I*_*t*_, *O*_*t*_ denote the forget gate, the input gate and the output gate, respectively; ${\tilde{C}}_{t}$ is a cell input activation vector; *C*_*t*_ is the cell status; *h*_*t*_ is the output vector of the LSTM cell; *x*_*t*_ is the input vector to the LSTM cell; *W*_·_, *U*_·_, and *b*_·_ are the weight matrices and bias vector parameters to be tuned during training, respectively; σ is the sigmoid activation function; and ° denotes the element-wise product.

To better deal with non-linearity in data, we can stack LSTM layers upon each other. In the context of our analysis, we built a three-layer stacked LSTM architecture: the first layer having 64 cells, the second 32, and the third 16. We applied a dropout rate of 0.3 after each LSTM layer, and we used the tanh activation function mainly due to its fast convergency properties (LeCun et al., 1998). We used Adam with a learning rate of 0.001 as the optimization algorithm, and we trained our network for 1200 epochs, applying early stopping to further avoid overfitting (Goodfellow et al., 2016). We initialized the weights of each LSTM layer with the Glorot uniform initializer (Glorot and Bengio, 2010), which in practice works well for deep networks (Calin, 2020).

We also considered a benchmark GAN, which architecture is similar to the one proposed by Zhang et al. (2019): we adopted LSTM as generator, and a 3-layer MLP as discriminator. As suggested by Zhang et al. (2019), we used Leaky ReLU as activation function for the hidden layers, dropout as regularization method, and Binary Cross Entropy (BCE) loss function. As in our proposed GAN, we chose Adam as optimization algorithm.

To compare the results of the considered models, we need to select a loss function. We selected three metrics: the Root Mean Squared Error (RMSE), the Mean Absolute Error (MAE), and the Mean Absolute Percentage Error (MAPE) to assess the performance of our models and measure the difference between real and predicted values. Considering *n* observations, their equations are given by:


$$\begin{array}{lll} RMSE & = & \sqrt{\frac{1}{n}\;\overset{n}{\underset{i=1}{\sum }}{\left({ŷ}_{i}-y_{i\;}\right)}^{2}}\;,\\ MAE & = & \frac{1}{n}\;\overset{n}{\underset{i=1}{\sum }}|{ŷ}_{i}-y_{i\;}|,\\ MAPE & = & \frac{100}{n}\;\overset{n}{\underset{i=1}{\sum }}\left|\frac{y_{i\;}-{ŷ}_{i}}{y_{i\;}}\right|, \end{array}$$

where *y*_*i*_ is the true value, and ŷ_*i*_ is the predicted value obtained with the selected model. In general, RMSE penalizes large errors more than the other considered metrics, being more sensitive to outliers; on the other hand, MAE is often considered an easy and interpretable metric to describe the average error committed by a model. Finally, MAPE expresses the committed error as a percentage, favoring the comparison with different data.

We split our data (4,149 observations per each stock, from January 3rd, 2005, to April 30th, 2021) into a training set and a test set at a ratio of 80–20 (i.e., the first 80% of the time series of each stock was used for training the models and the last 20% was used to evaluate their performance on out-of-sample data). The training set consisted therefore of 3,320 observations (from January 3rd, 2005, to January 18th, 2018), and the test set consisted of 829 observations (from January 19th, 2018, to April 30th, 2021). Multi-step forecasting was implemented using a rolling forecast approach, where the true realizations of the closing price were made available to the models after each forecast iteration.

ARIMAX models have been implemented using the statistical package gretl^3^, while their SVR backends, the RF Regressors, the LSTM models, the benchmark GAN, and our proposed GAN are built with Python^4^. We collected the results for single-step forecasts in Table 3 and those for multi-step forecasts in Table 4.

**Table 3:** Single-step forecast errors for ARIMAX, RF Regressor, LSTM, the benchmark GAN, and our GAN architecture.

**Single-step forecasts**										
**Stock**	**Metrics**	**ARIMAX-SVR**		**RF Regressor**	**LSTM**		**Benchmark GAN**		**Our GAN**	
		**(p,d,q)**	**Results**	**Results**	**Lags**	**Results**	**Lags**	**Results**	**Lags**	**Results**
ATL	RMSE	(5,1,0)	0.990	0.814 (0.060)	2	0.763 (0.015)	2	0.890 (0.120)	2	**0.631 (0.043)**
	MAE		0.820	0.543 (0.022)		0.501 (0.011)		0.694 (0.118)		**0.416 (0.029)**
	MAPE		4.871	3.034 (0.177)		2.779 (0.052)		3.821 (0.829)		**2.294 (0.134)**
AZM	RMSE	(5,1,0)	0.663	0.633 (0.084)	2	0.582 (0.004)	2	0.895 (0.149)	2	**0.541 (0.011)**
	MAE		0.530	0.460 (0.053)		0.403 (0.004)		0.788 (0.150)		**0.347 (0.003)**
	MAPE		3.544	2.900 (0.329)		2.603 (0.035)		5.306 (1.050)		**2.329 (0.028)**
BZU	RMSE	(4,1,0)	0.899	0.639 (0.038)	2	0.578 (0.006)	2	0.700 (0.106)	2	**0.506 (0.008)**
	MAE		0.796	0.479 (0.023)		0.426 (0.006)		0.560 (0.124)		**0.366 (0.006)**
	MAPE		4.128	2.496 (0.117)		2.225 (0.032)		2.951 (0.629)		**1.911 (0.030)**
ENEL	RMSE	(2,1,0)	0.427	0.467 (0.022)	2	0.266 (0.023)	2	0.352 (0.097)	2	**0.180 (0.013)**
	MAE		0.313	0.274 (0.021)		0.179 (0.017)		0.179 (0.017)		**0.120 (0.011)**
	MAPE		4.488	3.582 (0.017)		2.605 (0.213)		4.863 (0.635)		**1.797 (0.150)**
ENI	RMSE	(1,1,0)	1.390	1.874 (0.016)	2	1.231 (0.064)	2	1.093 (0.260)	2	**0.391 (0.073)**
	MAE		1.124	1.152 (0.014)		0.838 (0.033)		0.951 (0.258)		**0.280 (0.059)**
	MAPE		11.194	13.839 (0.144)		9.568 (0.441)		8.372 (2.065)		**2.688 (0.620)**
EXO	RMSE	(1,1,0)	3.533	4.410 (0.014)	2	2.662 (0.186)	2	2.749 (0.636)	2	**1.930 (0.113)**
	MAE		2.635	2.828 (0.011)		1.945 (0.136)		2.250 (0.565)		**1.403 (0.132)**
	MAPE		4.719	4.438 (0.028)		3.315 (0.214)		3.907 (0.975)		**2.454 (0.227)**
G	RMSE	(5,1,0)	0.458	0.400 (0.022)	2	0.390 (0.005)	2	0.448 (0.168)	2	**0.319 (0.020)**
	MAE		0.324	0.283 (0.010)		0.276 (0.007)		0.344 (0.157)		**0.227 (0.019)**
	MAPE		2.181	1.910 (0.069)		1.888 (0.042)		2.314 (1.013)		**1.548 (0.127)**
IP	RMSE	(1,1,0)	1.449	4.326 (0.012)	2	1.464 (0.010)	2	1.368 (0.193)	2	**1.098 (0.205)**
	MAE		1.096	2.179 (0.043)		1.015 (0.074)		1.078 (0.170)		**0.800 (0.124)**
	MAPE		3.795	5.896 (0.074)		3.286 (0.267)		3.607 (0.663)		**2.662 (0.330)**
MB	RMSE	(5,1,0)	0.305	0.289 (0.009)	2	0.282 (0.002)	2	0.352 (0.107)	2	**0.225 (0.003)**
	MAE		0.240	0.212 (0.004)		0.200 (0.003)		0.283 (0.108)		**0.164 (0.002)**
	MAPE		3.183	2.654 (0.081)		2.624 (0.028)		3.760 (1.485)		**2.146 (0.028)**
REC	RMSE	(2,1,0)	2.544	2.723 (0.014)	2	1.843 (0.122)	2	1.631 (0.452)	2	**1.029 (0.132)**
	MAE		2.223	1.725 (0.014)		1.384 (0.092)		1.250 (0.369)		**0.754 (0.130)**
	MAPE		6.231	4.066 (0.093)		3.559 (0.226)		3.296 (0.966)		**1.999 (0.328)**

*Text in bold denotes the best results (95% confidence level)*.

**Table 4:** Multi-step (5 days) forecast errors for ARIMAX, RF Regressor, LSTM, the benchmark GAN, and our GAN architecture.

**Multi-step forecasts**										
**Stock**	**Metrics**	**ARIMAX-SVR**		**RF Regressor**	**LSTM**		**Benchmark GAN**		**Our GAN**	
		**(p,d,q)**	**Results**	**Results**	**Lags**	**Results**	**Lags**	**Results**	**Lags**	**Results**
ATL	RMSE	(5,1,0)	1.784	1.027 (0.015)	5	1.080 (0.021)	2	2.319 (0.543)	2	**0.950 (0.027)**
	MAE		1.377	0.694 (0.009)		0.702 (0.014)		1.782 (0.424)		**0.620 (0.021)**
	MAPE		8.267	3.833 (0.052)		3.920 (0.074)		9.409 (2.393)		**3.287 (0.118)**
AZM	RMSE	(5,1,0)	1.776	0.977 (0.027)	5	0.930 (0.018)	2	2.274 (0.635)	2	**0.850 (0.024)**
	MAE		1.380	0.697 (0.013)		0.647 (0.013)		2.050 (0.540)		**0.588 (0.020)**
	MAPE		9.394	4.444 (0.088)		4.172 (0.083)		14.026 (2.809)		**3.844 (0.176)**
BZU	RMSE	(4,1,0)	1.799	0.906 (0.016)	5	0.886 (0.026)	2	2.444 (0.651)	2	**0.784 (0.030)**
	MAE		1.549	0.669 (0.007)		0.654 (0.018)		1.984 (0.603)		**0.572 (0.022)**
	MAPE		8.117	3.489 (0.039)		3.443 (0.095)		11.179 (4.202)		**2.951 (0.108)**
ENEL	RMSE	(2,1,0)	0.607	0.563 (0.005)	5	0.388 (0.016)	2	1.647 (0.622)	2	**0.268 (0.023)**
	MAE		0.449	0.357 (0.005)		0.267 (0.010)		1.283 (0.472)		**0.182 (0.020)**
	MAPE		6.379	4.829 (0.096)		3.871 (0.113)		20.652 (4.301)		**2.771 (0.279)**
ENI	RMSE	(1,1,0)	1.649	2.114 (0.013)	5	1.325 (0.081)	2	2.041 (0.624)	2	**0.888 (0.193)**
	MAE		1.200	1.366 (0.012)		0.909 (0.053)		1.418 (0.437)		**0.636 (0.134)**
	MAPE		12.514	16.028 (0.121)		10.288 (0.639)		12.156 (3.720)		**5.884 (1.164)**
EXO	RMSE	(1,1,0)	5.201	4.840 (0.013)	5	4.116 (0.071)	2	5.721 (1.044)	2	**2.955 (0.218)**
	MAE		3.691	3.363 (0.013)		3.047 (0.030)		4.776 (0.870)		**2.264 (0.258)**
	MAPE		6.664	5.461 (0.022)		5.195 (0.034)		9.061 (1.909)		**3.977 (0.461)**
G	RMSE	(5,1,0)	0.526	0.622 (0.018)	5	0.564 (0.004)	2	0.860 (0.338)	2	**0.489 (0.003)**
	MAE		0.369	0.441 (0.010)		0.385 (0.006)		0.721 (0.288)		**0.339 (0.010)**
	MAPE		2.445	3.000 (0.007)		2.641 (0.035)		4.763 (1.879)		**2.281 (0.076)**
IP	RMSE	(1,1,0)	2.057	4.363 (0.017)	5	2.557 (0.089)	2	2.866 (0.716)	2	**1.366 (0.155)**
	MAE		1.674	2.350 (0.013)		1.670 (0.033)		2.404 (0.603)		**1.007 (0.139)**
	MAPE		5.761	6.629 (0.028)		5.178 (0.080)		7.941 (1.989)		**3.577 (0.527)**
MB	RMSE	(5,1,0)	0.680	0.474 (0.020)	5	0.419 (0.002)	2	0.880 (0.566)	2	**0.361 (0.012)**
	MAE		0.508	0.335 (0.013)		0.289 (0.004)		0.665 (0.354)		**0.257 (0.011)**
	MAPE		6.738	4.416 (0.183)		3.836 (0.055)		7.059 (2.086)		**3.300 (0.143)**
REC	RMSE	(2,1,0)	3.742	3.231 (0.096)	5	2.356 (0.117)	2	4.736 (1.196)	2	**1.776 (0.261)**
	MAE		3.278	2.191 (0.065)		**1.625 (0.286)**		3.875 (1.047)		**1.403 (0.236)**
	MAPE		9.320	5.295 (0.155)		4.427 (0.174)		10.886 (2.780)		**3.713 (0.626)**

*Text in bold denotes the best results (95% confidence level)*.

In Tables 3, 4 we denoted, for each stock and metric, the best performance (lowest value) in bold. From Table 3, we can observe how the DCGAN architecture performed significatively better than the other considered models for all the 10 considered stocks. The same good performances are obtained when we tried to forecast the next week (i.e., the next 5 days) at once: indeed, as we can observe from Table 4, our GAN architecture again significatively performed better than its competitors for all the stocks and for all the metrics, except for the MAE of a single stock, where there was not a statistically significant difference. It is also interesting to note how our DCGAN performs with respect to the benchmark GAN. First, while the benchmark GAN results can be considered good when performing single-step forecasting, they get much worse in the multi-step scenario. On the other hand, our architecture is much more consistent. Second, both in the single-step and in the multi-step scenarios, the standard deviation of our GAN is much lower than that of the benchmark GAN, highlighting how the proposed architecture is stable and does not suffer much from unstable training, which is a well-known open problem in GANs training (Chen, 2021).

There is indeed an inherent stochastic component in the training process of deep learning models, mainly coming from the weight initialization scheme and the optimization process. We reported in tables the average mean of 10 independent runs of each model, and in brackets the standard deviation. For each stock, to confirm statistically significant differences in the metrics results, we ran independent *t*-tests with *p* <0.05.

We further compared the accuracy of the obtained forecasts using the Diebold-Mariano (DM) test (Diebold and Mariano, 1995). Considering two forecasts, the null hypothesis of the DM test is that they have the same accuracy (i.e., there are no differences in the forecasts), while the alternative hypothesis is that the two considered forecasts have different degrees of accuracy. We reported in Table 5 the results of the DM test for the considered models. We can observe that, at 95% confidence level, the null hypothesis of our GAN forecasts having the same accuracy with other models is rejected for all the scenarios, except for two cases in the multi-step comparison with RF Regressor and LSTM (highlighted in bold).

**Table 5:** Diebold-Mariano test for the single-step and multi-step forecasts.

**Stock**	**ARIMAX-SVR**	**RF Regressor**	**LSTM**	**Benchmark GAN**
	**Diebold-Mariano test single-step forecasts (** ***p*** **-value)**			
ATL	0	0	0	0
AZM	0	0	0.025	0
BZU	0	0	0	0
ENEL	0	0	0	0
ENI	0	0	0	0
EXO	0	0	0	0
G	0	0	0	0
IP	0	0	0	0
MB	0	0	0	0
REC	0	0	0	0
	**Diebold-Mariano test multi-step forecasts (** ***p*** **-value)**			
ATL	0	**0.2078**	0.0064	0
AZM	0	0	0.0085	0
BZU	0	0	0.0004	0
ENEL	0	0	0	0
ENI	0	0	0	0
EXO	0	0	0	0
G	0	0	**0.0562**	0
IP	0	0	0.0012	0
MB	0	0	0.0081	0
REC	0	0	0	0

*The forecasts obtained from each competitor model have been compared with those of our GAN. Text in bold denotes the non-rejection of the null hypothesis of the test (95% confidence level)*.

**Figure 5:**
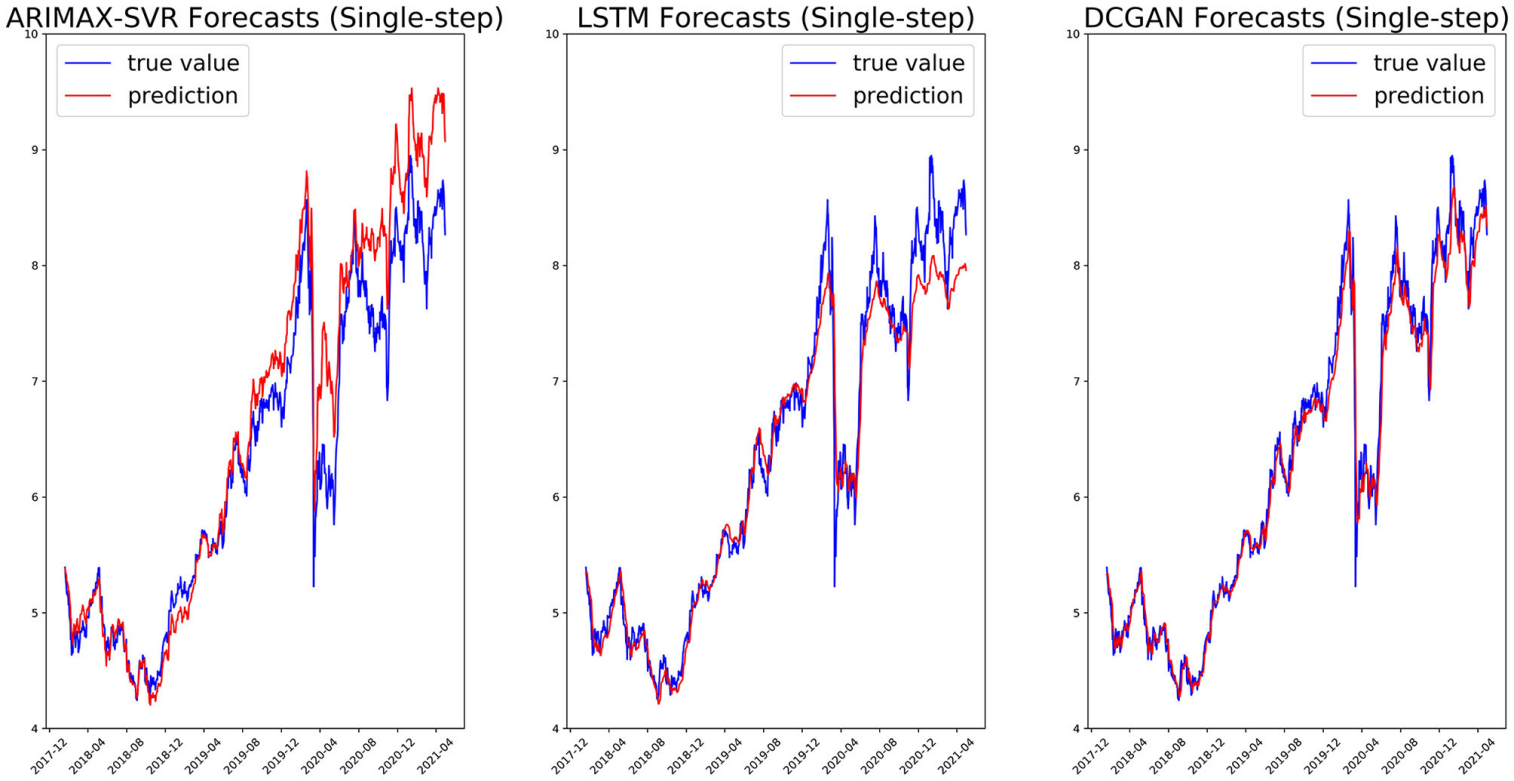
Single-step forecasts on the ENEL test set for ARIMAX-SVR **(left)**, LSTM **(center)**, and our GAN architecture **(right)**.

**Figure 6:**
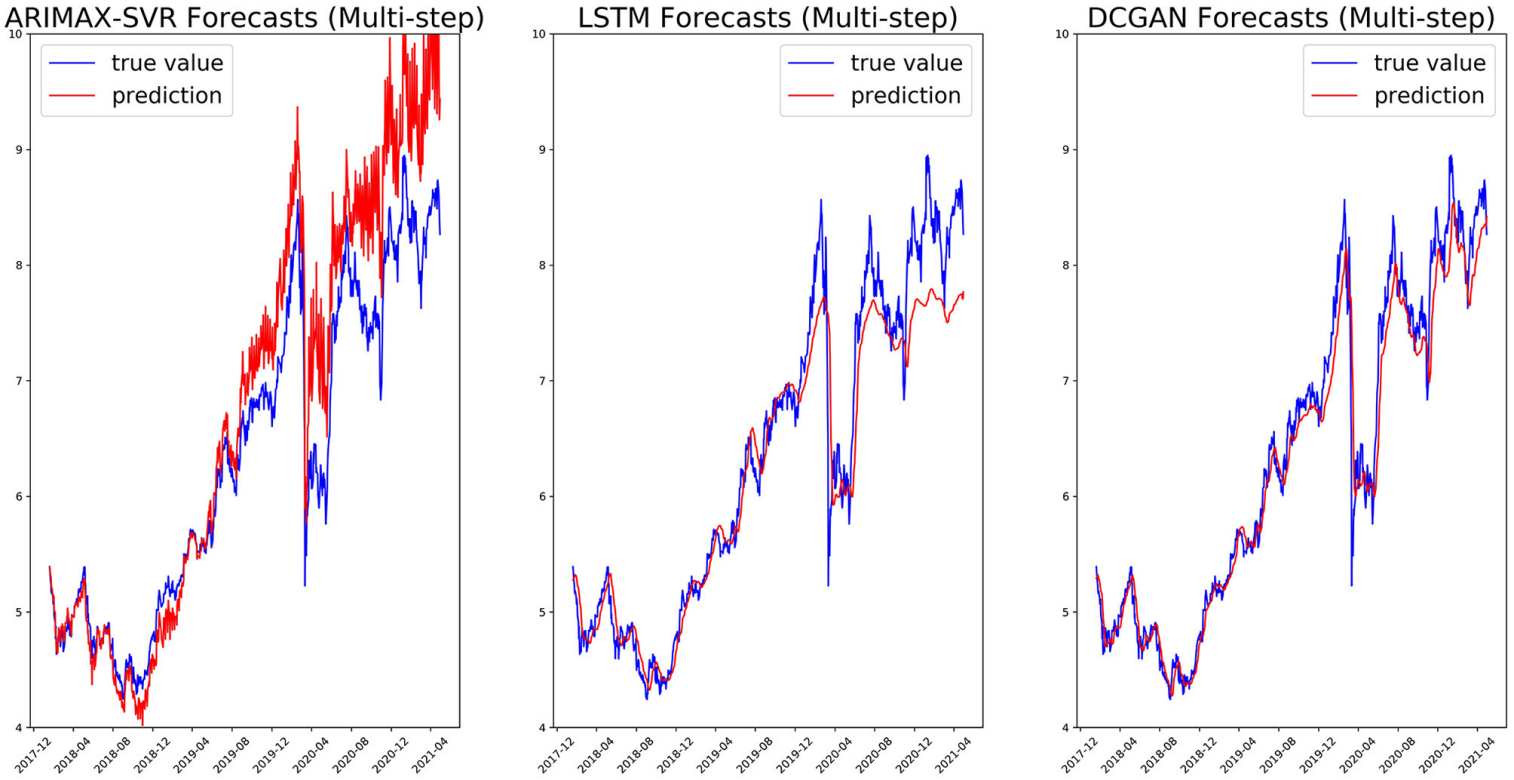
Multi-step (5 days) forecasts on the ENEL test set for ARIMAX-SVR **(left)**, LSTM **(center)**, and our GAN architecture **(right)**.

We, respectively, reported in Figures 5, 6 the single-step and multi-step forecasts of three considered models for a stock (ENEL).

The train-test split (80–20) has been chosen by comparing the model performances with those obtained with two others commonly used split percentages and considering the training set and test set representativeness. The results for one stock (EXO) are reported in Table 6, where the best performance for each metric (lowest value) is highlighted in bold. We can observe how the 80–20 split results in better performances across all the considered models and metrics.

**Table 6:** Train-test split results for one stock (EXO).

**Train-test split**	**Metrics**	**ARIMAX-SVR**	**RF regressor**	**LSTM**	**Benchmark GAN**	**Our GAN**
**Single-step forecast results on EXO**						
60–40	RMSE	6.001	20.838 (0.012)	12.237 (0.050)	21.225 (2.372)	19.987 (1.865)
	MAE	4.942	17.667 (0.013)	9.941 (0.061)	18.738 (2.695)	16.912 (1.860)
	MAPE	9.468	31.865 (0.025)	17.776 (0.142)	62.149 (8.831)	54.734 (4.003)
70–30	RMSE	8.121	12.290 (0.020)	4.562 (0.775)	4.419 (1.831)	3.936 (0.923)
	MAE	7.188	9.606 (0.011)	3.154 (0.584)	3.794 (1.495)	2.783 (0.728)
	MAPE	12.904	15.895 (0.029)	5.235 (0.930)	7.286 (2.951)	4.415 (1.000)
**80–20**	RMSE	**3.533**	**4.410 (0.014)**	**2.662 (0.186)**	**2.749 (0.636)**	**1.929 (0.112)**
	MAE	**2.635**	**2.828 (0.011)**	**1.945 (0.136)**	**2.250 (0.564)**	**1.402 (0.132)**
	MAPE	**4.719**	**4.438 (0.028)**	**3.315 (0.214)**	**3.906 (0.975)**	**2.454 (0.226)**
**Multi-step forecast results on EXO**						
60–40	RMSE	17.744	21.419 (0.008)	12.931 (0.039)	21.718 (2.617)	20.446 (0.665)
	MAE	15.593	18.334 (0.009)	10.415 (0.047)	19.722 (2.807)	17.553 (0.817)
	MAPE	28.859	33.269 (0.018)	18.541 (0.105)	64.813 (15.432)	53.491 (3.809)
70–30	RMSE	17.968	12.717 (0.018)	6.098 (0.084)	8.930 (3.041)	4.135 (0.813)
	MAE	16.006	10.143 (0.013)	4.368 (0.086)	7.073 (2.595)	3.285 (0.835)
	MAPE	27.758	16.953 (0.025)	7.254 (0.146)	12.863 (4.591)	6.131 (1.730)
**80–20**	RMSE	**5.201**	**4.840 (0.013)**	**4.116 (0.713)**	**5.721 (1.043)**	**2.955 (0.218)**
	MAE	**3.691**	**3.363 (0.013)**	**3.047 (0.030)**	**4.776 (0.869)**	**2.264 (0.258)**
	MAPE	**6.664**	**5.461 (0.022)**	**5.195 (0.034)**	**9.060 (1.909)**	**3.977 (0.461)**

*Text in bold denotes the best performance (99% confidence level)*.

For what concerns the execution times of the considered models, they vary greatly according to the model complexity: ARIMAX-SVR is the faster, while our proposed GAN is the slowest. A more detailed description, with the average execution times and the standard deviations for each model, is reported in Table 7.

**Table 7:** Average execution times of the considered models (10 runs on a single stock). We ran our experiments on a Microsoft Windows 10 (Version 21H1), with 11th Gen Intel(R) Core (TM) i7-1165G7 processor (2.80 GHz, 16.0 GB of RAM).

**Model**	**Time (in seconds)**	**Standard deviation**
**Single-step forecasts average execution time**		
ARIMAX-SVR	2.30	0.34
RF Regressor	7.49	1.06
LSTM	71.72	1.37
Benchmark GAN	162.63	5.21
Our GAN	2,394.60	17.16
**Multi-step forecasts average execution time**		
ARIMAX-SVR	2.38	0.05
RF Regressor	19.73	2.10
LSTM	113.71	2.11
Benchmark GAN	1,097.67	30.98
Our GAN	3,011.47	70.64

## Conclusions

In this paper, we proposed a DCGAN architecture with a CNN-BiLSTM generator and a CNN discriminator, with the goal of making accurate forecasts on the closing price of stocks. We compared its performance with those of four benchmark models (ARIMAX-SVR, Random Forest Regressor, LSTM, and a benchmark GAN) over 10 different stock time series. Both in the single-step and in the multi-step scenario, the results of our proposed architecture were always better (or on par, for a single metric in a single case) than the results of the benchmark models, suggesting that financial time series forecasting may benefit from employing GANs.

Despite having obtained promising results and a stable architecture, training GANs remains a difficult task due to the need of tuning many hyperparameters while keeping a balance between the generator and the discriminator network. Future research should move in the direction of finding a more systematic way to perform parameter tuning, as well as exploring other architecture configurations.

It should also be mentioned that we considered a fixed test set and evaluated all the models on it. An interesting extension of our work would be to compare the performances in other settings: for example, exclude crisis periods (such as the COVID-19 crisis), consider different forecasting horizons, and different time granularities.

## Data Availability Statement

The original contributions presented in the study are included in the article/supplementary materials, further inquiries can be directed to the corresponding author/s.

## Author Contributions

AS: conceptualization, methodology, software, validation, analysis, investigation, writing, and visualization.

## Conflict of Interest

Author AS was employed by ALBERT Inc.

## Publisher's Note

All claims expressed in this article are solely those of the authors and do not necessarily represent those of their affiliated organizations, or those of the publisher, the editors and the reviewers. Any product that may be evaluated in this article, or claim that may be made by its manufacturer, is not guaranteed or endorsed by the publisher.

## Footnotes

^1^Technically, even only finding these points is NP-hard.

^2^Given any $x_{1},x_{2}\in R^{n}$, a function *f*:*R*^*n*^→*R* is called 1-Lipschitz continuous if it satisfies |*f*(*x*_1_)−*f*(*x*_2_)| ≤ ||*x*_1_−*x*_2_||.

^3^Version: 2020b.

^4^Version: 3.7.3, run on an Anaconda platform.
